# Droplets for Sampling and Transport of Chemical Signals in Biosensing: A Review

**DOI:** 10.3390/bios9020080

**Published:** 2019-06-20

**Authors:** Shilun Feng, Elham Shirani, David W. Inglis

**Affiliations:** 1 School of Engineering, Macquarie University, Sydney, NSW 2109, Australia; elham.shirani-faradonbeh@students.mq.edu.au; 2 ARC Centre of Excellence for Nanoscale BioPhotonics (CNBP), Macquarie University, Sydney, NSW 2109, Australia

**Keywords:** microfluidic probe, droplet, sampling, Taylor dispersion

## Abstract

The chemical, temporal, and spatial resolution of chemical signals that are sampled and transported with continuous flow is limited because of Taylor dispersion. Droplets have been used to solve this problem by digitizing chemical signals into discrete segments that can be transported for a long distance or a long time without loss of chemical, temporal or spatial precision. In this review, we describe Taylor dispersion, sampling theory, and Laplace pressure, and give examples of sampling probes that have used droplets to sample or/and transport fluid from a continuous medium, such as cell culture or nerve tissue, for external analysis. The examples are categorized, as follows: (1) Aqueous-phase sampling with downstream droplet formation; (2) preformed droplets for sampling; and (3) droplets formed near the analyte source. Finally, strategies for downstream sample recovery for conventional analysis are described.

## 1. Introduction

Biosensors have broad applications in drug discovery, medical diagnostics [1,2], environmental monitoring and food safety [3]. The concentration of specific analytes and their reaction kinetics can be identified in biosensors [4,5]. Biosensors using microdroplets have offered reconfigurability and flexibility and limited contamination during sample preparation and analysis [4]. In this review, we focus on the use of droplets to improve the temporal, spatial and chemical resolution of biosensing measurements by compartmentalizing samples during transport from the sampling site, to the analytical site. In doing so, it is possible to reduce Taylor dispersion.

**Figure 1:**
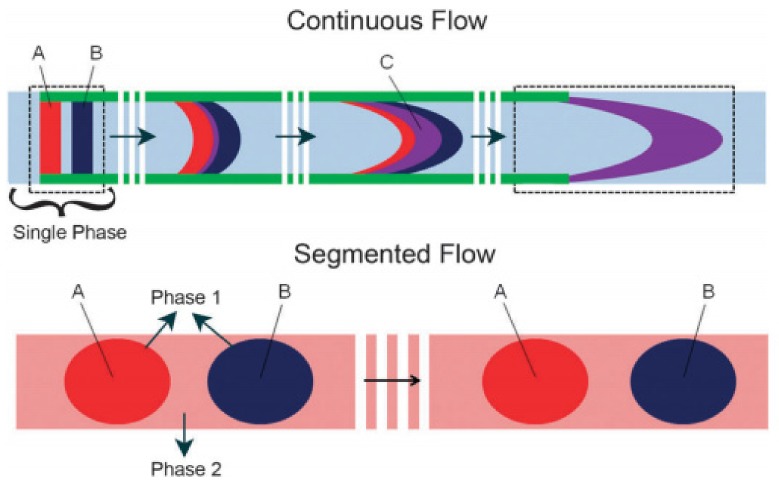
Schematic representation of using segmented flow to avoid the Taylor dispersion. (The vertical white lines represent the passage of space and time (Reprinted with permission from Reference [8]. Copyright 2011 Royal Society of Chemistry).

In 1953, Taylor dispersion was described as “the combined action of molecular diffusion and the variation of velocity over the cross section” [6]. Because of this dispersive phenomena, the distance that chemical signals (variation in concentration with time and space) or analytes can be transported in the continuous phase is severely limited [6,7]. The top part of Figure 1 shows pulses of imaginary chemical A and chemical B. Initially, the pulses are separated from one another, but after a short distance the chemical pulses have merged and spread out [8]. In contrast, the bottom part of Figure 1 [8] shows that pulses of chemical A and B, when contained in droplets are not dispersed or merged after transport. The sampling of tiny volumes of aqueous body fluids and their transport to systems for precise detection or quantification is an ongoing area of activity in biomedical research [9,10,11,12,13,14,15,16,17,18,19,20,21,22,23,24,25].

The chemical content of a sample taken from a biological system may change, depending on when and where the sample is taken. For example, changes in neurotransmitter concentrations in the extracellular space around synapses are known to happen in milliseconds to seconds [26,27,28,29]. It is also well known that several neurotransmitters coexist in a given synaptic region, and that they can be released at different times [28,29]. It has been shown by Bert [30] that glutamate changes occurring in 1 min can be completely dampened when samples are pooled, as opposed to discretized.

It is reported that temporal resolution for conventional high-performance liquid chromatography (HPLC) is minutes [31]. Such time scales make it impossible to quantify analyte concentrations that change rapidly accurately. Droplet-based approaches can be used to effectively avoid dispersion and improve temporal resolution by capturing and storing events that occur too quickly for a particular analytical method [32,33,34,35,36,37]. Microdialysis sampling, coupled with droplets and direct infusion mass spectrometry was used for acetylcholine monitoring [38] with intervals of just a few seconds. Monitoring of real-time streptavidin–biotin binding kinetics was also achieved using droplet microfluidics integrated with confocal spectroscopy [39]. Srinivasan et al. [40] reported the integration of optical absorbance measurements with droplet-based microfluidics for the detection of glucose using glucose oxidase in less than 40 s. The coupling of a digital droplet-based microfluidic device to surface plasmon resonance (SPR) imaging has also been demonstrated [41,42].

Water-in-oil droplets are not perfect containers. Water and small molecules move through the water-oil interface at a non-zero rate [43]. Surfactants at the interface may form micelles, leading to another mechanism for analytes to escape droplets. Air bubbles have been used as separators between aqueous slugs to reduce cross contamination [44], and leakage is suspected to be worse in the corners of rectangular channels [45].

Conventional sampling tools, which can undertake a continuous sampling of body fluid, cannot sample and transport rapid changes of chemical signals from the insertion point to an analytical instrument without signal distortion, because of the Taylor dispersion phenomenon. Similarly, the same signal distortion problem also occurs when delivering sequences of different drugs to the injection point. A real-time analytical chemistry lab, small enough to fit inside the brain of a mouse does not exist. However, we can achieve similar analytical aims if we can digitize the liquid environment from precise locations within an organism at precise times using water-based liquids, carried by an immiscible oil in a hydrophobic channel. In this paper, we review state-of-the-art low-volume sampling probes that use droplets to transport signals for downstream analysis. For these sampling tools, hydrophobic and hydrophilic surfaces are used to control the movement of liquids.

## 2. Theory

### 2.1. Taylor Dispersion

Taylor dispersion acts to enhance diffusion, which can reduce the temporal and chemical resolution of biosensors [46,47,48], DNA analysis [49,50,51], mass spectrometry [52,53,54], surface patterning [55,56,57,58] and other applications. It results from the interaction of convection and diffusion within a pipe or channel.

Convection is the transport of fluid axially in the flow direction. The typical Poiseuille laminar flow in a low Reynolds number channel has its maximum flow velocity in the centre of the channel and decreases smoothly to zero at the walls. Volumes of fluid near the centre of the channel will move much faster than those near the walls. A group of molecules, initially near one-another is, thus, spread out. Diffusion makes the problem worse. Diffusion is driven by a gradient in the chemical concentration of the diffusing species [59]. When considering a group of molecules forming a pulse of an analyte, the sharper the gradient constituting that pulse, the more rapidly it disappears.

The effective diffusion in a capillary was described by Sir Geoffrey Taylor and R Aris [60], it is as the sum of conventional axial diffusion (*D*) and the Taylor dispersion coefficient. Together they are given an equation for the effective diffusion coefficient in a capillary known as the Taylor-Aris dispersion coefficient [61,62]:

(1) $$D_{eff}=D\left(1+\frac{1}{48}Pe_{d}{}^{2}\right),$$

where $Pe_{d}$ is the Peclet number, and is the ratio of convective fluxes to diffusive fluxes in the system. It can be defined as $Pe_{d}=2r\bar{V}/D$ where *r* is the channel radius, and $\bar{V}$ is the average value of the velocity in the Poiseuille flow. Combining the two makes it clear that when the average velocity is greater than $2\sqrt{3}D/r$, advection is the dominant cause of diffusion and dispersion. For a typical small molecule of Sucrose chemicals (D_Sucrose_ = 500 μm^2^/s) in a 100-um-wide channel, Taylor dispersion dominates when the average velocity is above 17 μm/s. This very low velocity highlights the futility of moving chemical signals in microchannels using continuous flow.

### 2.2. Biosensing with a Chemical, Spatial and Temporal Resolution

The sampling and delivery of tiny amounts of body fluids for accurate analysis is of great interest for fundamental biological studies, diagnostics, and therapeutics [22,25]. Specific and responsive signals, derived from the patient’s body can help the study of fundamental biological processes, and optimize the use of medical therapies by allowing them to be more accurately dosed and more precisely targeted [63,64]. Effective droplet generation transport and analysis may allow advances in biosensing through improved chemical, spatial and temporal resolution. These, in turn, can be used for a wide range of applications.

An in vivo measurement system should be concerned with three parameters: Analytical performance, spatial resolution, and temporal resolution. Analytical performance refers to a variety of measurement parameters. These include the minimum concentration that can be detected (limit of detection), the smallest difference between two samples that can be resolved (resolution), and the ability of the measurement to respond solely to changes in the target analyte concentration (specificity or selectivity).

Spatial resolution is a well-understood concept in imaging, where the term voxel refers to a 3D volume over which the information is averaged. The same principle can be applied to chemical sampling, where higher spatial resolution gives more localized information. Given that chemicals can permeate surrounding tissue, and that signals produced in one place spread, and, therefore, decrease in concentration, we would also expect a higher spatial resolution to enable the detection of more rapid changes in analyte concentrations from smaller sources. The cross-section of the probe in contact with the tissue is a primary determinant of spatial resolution. However, the volume of sample extracted will also influence the spatial resolution. Drawing a large volume with a very fine probe will average a larger chemical voxel, than drawing a small volume with the same size probe [65,66,67,68].

Temporal resolution refers to the time taken for the measured value to change in response to a step-change in the sample. This change may be fit to a single exponential, which allows for an easily defined time constant [69]. Temporal resolution may also be defined as *t_res_ =* Ø/*f*, where *f* is the sampling frequency or rate (Hz), and Ø is the number of plugs required to observe a change (from 10 to 90% of a concentration step) [70]; In another example [10], Ø was defined as the number of plugs (or samples) needed to exchange 95% of molecules of interest.

The response time of a complete system may also be limited by reaction kinetics at a sensor surface, but in this review, we are interested in the fundamental response time of the sampling process. This time delay is created by the movement of molecules from the signal source to the location of droplet break-up. Once in the droplets, we assume the droplet contents are fully mixed, and we ignore any further chemical reactions or changes that might occur inside or between the droplets. For a probe that extracts volume from the source, this time is approximately equal to the volume of liquid between source and droplet, divided by the volume flow rate. The temporal resolution can be reduced by extracting more fluid from the tissue, but this may damage or interfere with normal physiology.

For a probe that relies on diffusion across a membrane (microdialysis probes, for example), the speed of molecular transport is proportional to the analyte gradient and permeability of the membrane [71]. A device could sample a small percentage of the analyte with a high perfusion rate, achieving greater temporal resolution. However, capturing a high percentage of the source signal requires the concentrations on either side of the membrane to approach equilibrium, and, thus, a lower perfusion rate [72], and lower temporal resolution.

In a droplet system, we should consider the sampling rate and its relationship to the rate change of the thing being measured. Electrical engineers have addressed this problem through frequency analysis and arrived at a sampling theorem, which states that the sampling rate (samples per second) should be twice as fast as the fastest changing component of the signal, the Nyquist rate.

### 2.3. Interface Forces

Biological and chemical signals are typically generated in a continuous aqueous environment, and most analytical processes take the same fluid phase as inputs. Transporting signals from one location to another requires the digitizing of liquid packages at one end of the channel, analogous to an analog to digital conversion in electronics. At the receiving end of the channel, the reverse may happen. Here, the oil phase is removed in a process analogous to digital to analog (D2A) conversion. This packaging and unpackaging must overcome interfacial forces, the primary one being Laplace or bubble pressure. This pressure is controlled by three parameters: Surface tension, contact angle and hydraulic diameter.

Surface tension is the first parameter that can affect the Laplace pressure. It is defined pragmatically as: If a line is drawn on the surface of an interface, then one can determine the equilibrium state by assuming that the molecules on one side of the line exert a force **τ** per unit length of the line on the molecules on the other side. The **τ** will be the surface tension, and it is directed tangent to the surface [73]. Any work done against this force will increase the surface energy of the system. Fluid interfaces minimize their energy by taking shapes that minimize their surface area. These shapes can generate pressure differences across the interface. This pressure is referred to as the Laplace or bubble pressure and is given by the following equation [73]:

(2) $$\Delta P=P_{inside}-P_{outside}=\tau \left(\frac{1}{R_{1}}+\frac{1}{R_{2}}\right),$$

where *R*_1_ and *R*_2_ are the principal radii of curvature and τ is the surface tension of the aqueous/oil interface. For spherical droplets, *R*_1_ and *R*_2_ are the same. Therefore, the Laplace pressure can be defined as [73]:

(3) $$\Delta P=\frac{2\tau }{R}.$$

For an interface that is bounded by a solid surface, such as a microchannel or membrane pore, the Laplace pressure will relate to the contact angle as [73]:

(4) $$\Delta P=\frac{2\tau }{R^{\prime }cos\theta },$$

where $R^{\prime }$ is the hydraulic diameter of the structure containing the interface, and θ is the contact angle for the two fluids at the solid surface. This pressure must be overcome to create droplets, and it can be used, as we shall see later, to control the movement of certain phases in a two-phase system [74,75,76,77,78].

## 3. Sampling Devices

In this review paper, we survey examples where droplets have been applied to the delivery and/or sampling of chemical signals. The work is divided into three categories: Aqueous phase sampling with downstream droplet formation; preformed droplets for sampling; and droplets formed near the analyte source.

### 3.1. Aqueous-Phase Sampling with Downstream Droplet Formation

This category is characterized by an aqueous phase sampling probe that draws a sample from within the tissue, then transports it to a microfluidic device outside the tissue. The sampled fluid is then segmented at the external microfluidic devices. Methods of obtaining a sample that we consider here are: Diffusion through a membrane (dialysis probe) and direct fluid extraction (push-pull cannula, push-pull microfabricated sampling probe, hydrophilic capillary tube).

#### 3.1.1. Diffusion through a Membrane

Microdialysis is widely used as the sampling probe for in vivo monitoring [79], clinical studies [80,81,82] and pharmacokinetics [83]. However, the drawback of this probe is that it has a large sampling surface. The membrane is typically over 2 mm long and more than 200 μm in outer diameter. This large area limits spatial resolution.

**Figure 2:**
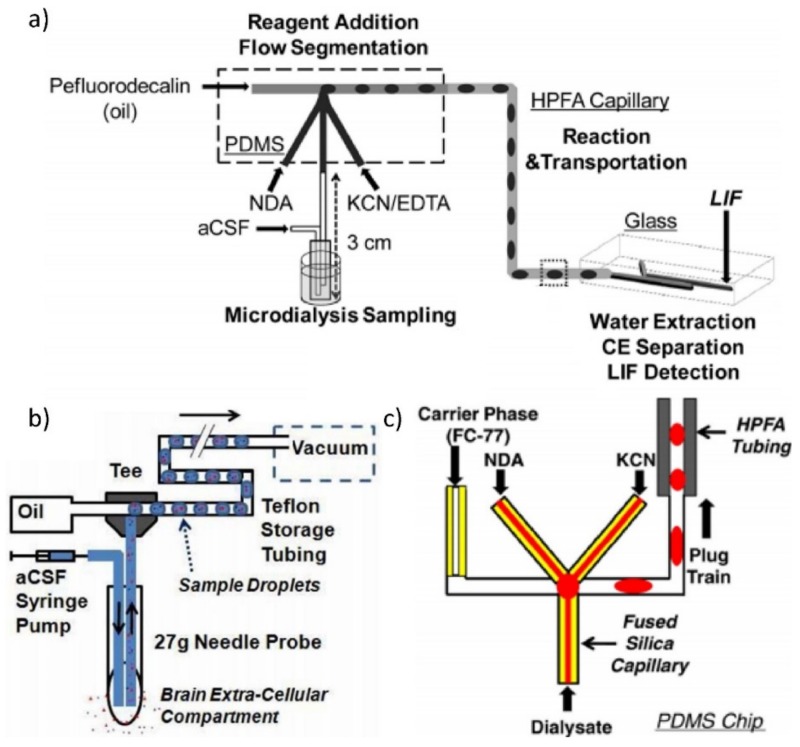
Continuous phase sampling with downstream droplet formation methods. (**a**) A microdialysis probe is used for sampling, coupled with a droplet generation chip to transport chemicals to a CE system (Reprinted with permission from Reference [85]. Copyright 2009 American Chemical Society); (**b**) research on low-flow push-pull probe system worked with a droplet system (Reprinted with permission from Reference [70]. Copyright 2011 American Chemical Society); (**c**) a capillary is used as the sampling probe, and attached to a droplet generator (Reprinted with permission from Reference [88]. Copyright 2011 Springer Nature).

A modern trend is to apply microdialysis in various clinical situations, such as monitoring concentrations of glucose, lactate, glutamate, and urea [84]. The microdialysis probe makes it possible for sampling to be done frequently without any loss of the volume from the tissue. Figure 2a shows a microdialysis probe coupled to a droplet generation chip to transport chemical signals to a distant capillary electrophoresis system [11,85]. The device has been used for sampling of neurotransmitter signals in a rat brain [86]. It has also been applied to an immobile live animal [87].

#### 3.1.2. Direct Fluid Extraction 

In this method, a buffer is continuously infused (pushed) into the tissue through one tube; while the sample is withdrawn (pulled) from a second tube that may be parallel or concentric with the infusion tube. These push-pull sampling systems are typically assembled by hand from capillary tubes and have been used in the brain since 1961 [89,90]. As above, the sample is segmented on a separate device that is a short distance downstream.

A temporal response of 5 min was achieved with low flow push-pull perfusion combined with off-chip fraction collection and analysis by capillary electrophoresis [68]. Lower temporal response of 16 s [91] and 45 s [92,93] was also achieved by coupling low-flow push-pull perfusion on-chip with CE for detection of samples from the eye and brain, respectively. Low-flow push-pull perfusion using a sampling probe with a smaller dead volume can be coupled with the segmented flow to achieve 7 s temporal response and spatial resolution of 0.016 mm^2^ in vivo [70] (Figure 2b). By further miniaturizing the probe inlet from 20 µm to a 10 µm, and reducing the dead volume, the authors showed in vitro sampling with 200 ms response time [70]. Figure 2c shows a probe with a 0.5 mm probe combined with a downstream droplet generator [88].

Very recently, van den Brink et al. demonstrated a microfabricated silicon-based push-pull probe with a 1 cm long probe and integrated droplet generation structure [94]. This device showed a temporal resolution of a few seconds and a sampling area of just 0.004 mm^2^. The device was used to record the glutamate level in the sensorimotor cortex of a mouse brain experiencing targeted electrical stimulation.

### 3.2. Preformed Droplets for Sampling

This section summarizes sampling methods that use pre-formed droplets. Song et al. used a hydrophilic capillary tube to sample the changing concentration solution of CaCl_2_ (0.2–0.4 μL/min, outside the chip. This hydrophilic capillary meets a hydrophobic channel carrying assay droplets. The sample is merged with the assay droplets for downstream analysis. The cross-section is 100 µm by 100 µm, but the length of the sampling capillary is unclear [95]. This length of capillary will cause dispersion.

**Figure 3:**
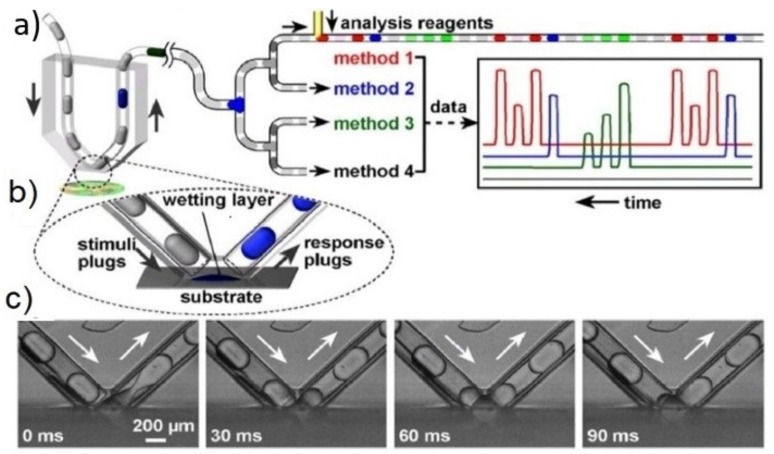
Pre-formed droplets are used to stimulate and extract analyte from a substrate, such as cell culture. (**a**) schematic of a device in operation; (**b**) an opening in the chip allows for droplet to merge with the hydrophilic substrate; (**c**) time-lapse bright-field images (side view) of the droplet extracting contents at 0 ms, 30 ms, 60 ms and 90 ms. (Reprinted with permission from Reference [10]. Copyright 2008 National Academy of Sciences, USA).

To solve the Taylor dispersion problem, Chen and Drew [96] and Chen et al. [10] brought droplets to the sampling site. Chen and Drew [96] proposed a microdialysis device where a pre-formed droplet passes a semi-permeable membrane. While on the membrane, analytes diffuse into the droplet. The droplet then moves downstream for analysis. D. Chen built a very similar system in the same year [10]. This approach, shown in Figure 3a–c, is used for sampling and/or introducing matter (stimulating) a planar environment, such as cell culture. A hydrophobic channel carrying oil is exposed on one side, to cells by clamping the device against a flat surface. Droplets are generated upstream of the interaction side. When those droplets reach the cells, they briefly make fluidic contact, exchange molecules, and are then carried away by the continuous oil phase.

The spatial resolution of the device was around 0.08 mm^2^ and was set by the size of the opening. In this work, the authors sampled at one droplet per second and showed >95% change in signal in just two droplets. The volume of each slug was 30 nL. The temporal response is not limited by Taylor dispersion because the sampling probe length is almost 0 (there is virtually no dead volume in the system). It should be noted that contamination of the tissue sample is quite possible as the channel was under positive pressure and had to be clamped to the tissue culture to prevent oil and buffer droplets from leaking into the tissue culture.

### 3.3. Droplets Formed near the Analyte Source

Several researchers have developed methods that reduce the distance over which dispersion may occur by forming droplets very near to the signal source. We can further categorize the examples by considering whether a membrane or other barrier exists between the microfluidic channel and the tissue to be sampled. 

#### 3.3.1. Droplets Formed near a Source without a Barrier

**Figure 4:**
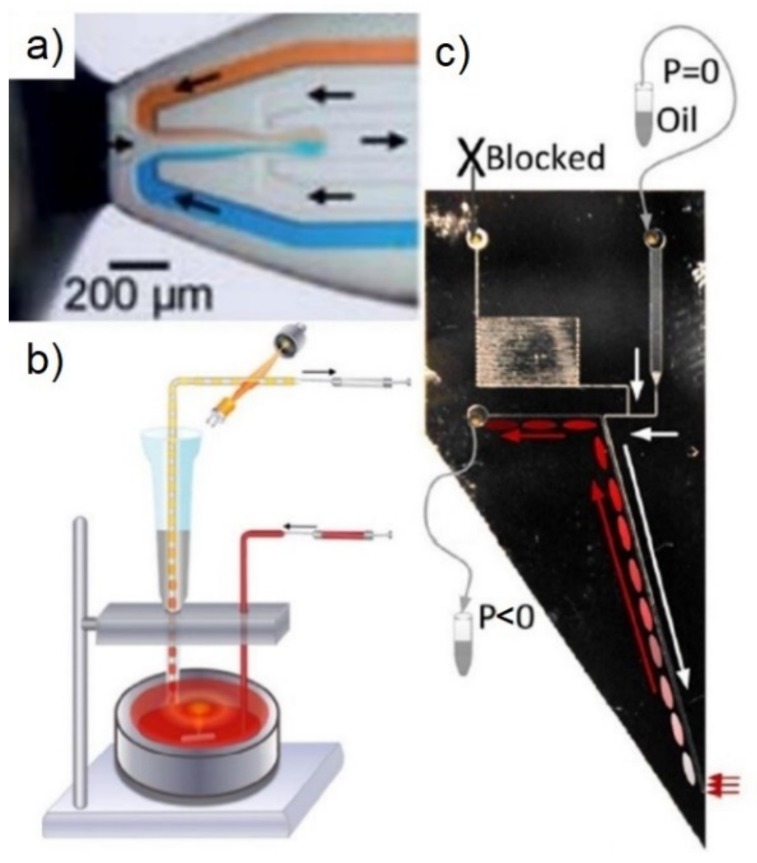
Methods that form droplets near the analyte source: (**a**) The middle channel is under negative pressure, sucking sample in through the device tip [97] (reprinted with permission. Copyright 2010 Royal Society of Chemistry); (**b**) droplet generated at the tip as co-flow. A Simple demonstration of sampling at the tip for 3-bromopropan-1-ol detection (reprinted with permission from Reference [98]. Copyright 2014 American Chemical Society); (**c**) sampling probe from Feng et al. which uses a microfabricated hydrophilic barrier (Reprinted with permission from Reference [69]. Copyright 2017 AIP Publishing LLC).

Figure 4a shows a device that has three channels; negative pressure is applied to the middle channel, generating enough force to draw the sample into the device and form droplets. Other channels supply aqueous sheath flow and a continuous phase [97]. Figure 4b illustrates a sampling probe made using a pipette tip and a concentric co-flowing oil. With carefully balanced pressures, droplets are formed at the tip and drawn into the Teflon tube [98]. MilliDrop (Paris France) is a commercial product that uses a similar approach, along with an air droplet to sample liquids from by dipping the tip in and out of the sample liquid.

#### 3.3.2. Droplets Formed at the Source with a Hydrophilic Barrier

Examples included in this section have implemented some form of Laplace-pressure-barrier to allow water to pass in or out of a hydrophobic channel, while retaining the oil-based continuous phase. Laplace pressure, known as bubble pressure in membrane science, is the pressure difference across a curved liquid interface. It manifests as a barrier to the ingress of air or oil into an already wet hydrophilic membrane.

Our group has fabricated a silicon device for droplet-based sampling [69]. Figure 4c shows this device, which only has 2.8 pL of dead volume between the channel and the exterior of the device. Hydrophilic structures, at the tip, contain this dead volume, and generate the surface tension barrier [69]. The device has been applied for on-site sampling and quantitative detection of Hydrogen Peroxide (H_2_O_2_). H_2_O_2_ samples were drawn into the device and immediately merged with assay droplets for reaction and downstream detection [99]. 

A hydrophilic membrane integrated within a planar microfluidic device has also been achieved. In this work, water in oil droplets is transported through a hydrophobic channel to a droplet exit port. The port is created by sealing a small section of the channel with a hydrophilic membrane. The wetted membrane resists penetration by the oil phase, but allows droplets to exit the channel [100]. Droplet delivery was demonstrated, but sampling was not.

## 4. Droplet Extraction for Downstream Analysis

A range of on-chip droplet-based detection and analysis methods are described in Qun Fang Group’s review [101]. However, many sophisticated chemical analysis techniques are not readily compatible with a sequence of droplets in a microchannel. Therefore, in this section, we review methods for extracting the transported samples so that they can be subjected to more conventional analytical methods. Three strategies are listed below that serve to remove the continuous phase that separates the droplets. These methods leave droplets accessible for further detection [21,102]. They are: Evaporation of the continuous phase; an oleophilic membrane to selectively extract the continuous phase; and using negative pressure at a hydrophobic-hydrophilic interface with extract the continuous phase.

**Figure 5:**
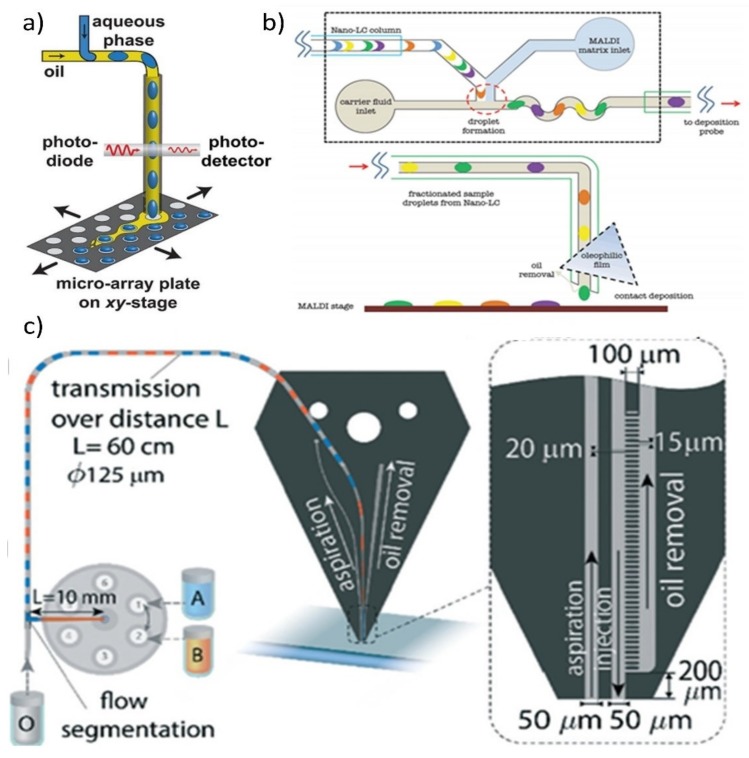
Off-chip water-based droplet extraction methods. (**a**) Schematic of droplet creation and spotting on a MALDI plate, where the hydrophobic carrier (oil) will evaporate quickly (Reprinted with permission from Reference [103]. Copyright 2013 American Chemical Society); (**b**) schematic of the device that used oleophilic oil film to extract the oil continuous phase and left the aqueous phase to the MALDI plate (Reprinted with permission from Reference [106]. Copyright 2013 PLOS ONE); (**c**) the developed microfluidic probe (MFP) system (Reprinted with permission from Reference [7]. Copyright 2016 Royal Society of Chemistry).

Some oils evaporate readily. In Figure 5a, the aqueous droplet phase is placed one by one on a Matrix Assisted Laser Desorption/Ionization (MALDI) plate together with the volatile perfluorinated oil (Perfuorohexane). Evaporation of both phases happens before the plate is loaded into the MALDI mass spectrometer. The evaporation time can be completed within less than 60 s, or accelerated to less than 5 s with a flow of nitrogen gas [103]. It is also possible to form segmented flows (slugs) in liquid-gas microfluidic systems [104]. Here the water droplets can easily be isolated through evaporation; however, the droplets themselves are less stable [105].

The oil phase can be absorbed by an oleophilic and hydrophobic membrane. Figure 5b shows that an oleophilic membrane made of Polytetrafluoroethylene (PTFE) was used to absorb and extract the continuous phase [106]. The aqueous phase was left suspended and placed one drop at a time on the hydrophilic part of a MALDI plate.

The oil phase can also be removed by negative pressure with the assistance of a hydrophobic-hydrophilic interface. Figure 5c shows a microfluidic probe (MFP) system for writing chemical patterns [7,107]. This device uses segmented flows to allow different chemicals to be delivered through the same orifice. Droplets were generated using a standard T-junction, and transported to the probe tip. Negative pressure was provided at the oil removal channel of the probe’s tip to remove the oil, while the small hydrophobic features function to retain the aqueous phase, due to the Laplace pressure. The water-based liquids are, thereby, expelled.

## 5. Conclusions

Point of care diagnostic devices reduce the distance that chemical signals must travel, and therefore, the dispersion, but for typical microchannels, it takes just millimeters for dispersion to be significant [69]. Furthermore, not all sensors can be brought to the sampling site. There have been many reviews of droplet-based microfluidics devices and droplet analysis methods. We focus on microfluidic slugs or droplets as a method of transporting chemical signals to overcome Taylor dispersion, and the devices use to capture those signals. We have surveyed examples of chemical signal sampling and delivery and classified them into three types: Aqueous phase sampling with downstream droplet formation; Preformed droplets for sampling; and Droplets formed near the analyte source. We pay particular attention to the temporal and spatial resolution of each system, and explain the features that limit each of these parameters. Strategies for downstream analysis have also been listed. We hope this review can help to broaden the use of droplet-based sampling probes for biochemical applications, enabling higher resolution study of fundamental processes.
